# Transcriptome analysis of wheat inoculated with *Fusarium graminearum*

**DOI:** 10.3389/fpls.2015.00867

**Published:** 2015-10-20

**Authors:** Mustafa Erayman, Mine Turktas, Guray Akdogan, Tugba Gurkok, Behcet Inal, Emre Ishakoglu, Emre Ilhan, Turgay Unver

**Affiliations:** ^1^Department of Biology, Faculty of Science, Mustafa Kemal UniversityHatay, Turkey; ^2^Department of Biology, Faculty of Science, Çankırı Karatekin UniversityÇankırı, Turkey; ^3^Department of Field Crops, Faculty of Agriculture, Ankara UniversityAnkara, Turkey; ^4^Department of Agricultural Biotechnology, Faculty of Agriculture, Siirt UniversitySiirt, Turkey

**Keywords:** early response, *Fusarium graminearum*, gene expression profiling, microarray, *Triticum aestivum* L

## Abstract

Plants are frequently exposed to microorganisms like fungi, bacteria, and viruses that cause biotic stresses. *Fusarium* head blight (FHB) is an economically risky wheat disease, which occurs upon *Fusarium graminearum* (Fg) infection. Moderately susceptible (cv. “Mizrak 98”) and susceptible (cv. “Gun 91”) winter type bread wheat cultivars were subjected to transcriptional profiling after exposure to Fg infection. To examine the early response to the pathogen in wheat, we measured gene expression alterations in mock and pathogen inoculated root crown of moderately susceptible (MS) and susceptible cultivars at 12 hours after inoculation (hai) using 12X135K microarray chip. The transcriptome analyses revealed that out of 39,179 transcripts, 3668 genes in microarray were significantly regulated at least in one time comparison. The majority of differentially regulated transcripts were associated with disease response and the gene expression mechanism. When the cultivars were compared, a number of transcripts and expression alterations varied within the cultivars. Especially membrane related transcripts were detected as differentially expressed. Moreover, diverse transcription factors showed significant fold change values among the cultivars. This study presented new insights to understand the early response of selected cultivars to the Fg at 12 hai. Through the KEGG analysis, we observed that the most altered transcripts were associated with starch and sucrose metabolism and gluconeogenesis pathways.

## Introduction

Along with rice and maize, bread wheat (*Triticum aestivum* L.) is one of the staple crops, being an important food in human diet. Its worldwide production is approximately 671 million tons per annum in 215 million Ha areas (Baloglu et al., 2014b; Inal et al., 2014; Okay et al., 2014). Wheat faces intensive biotic and abiotic stresses during its growth period. It was estimated that 10–16% of yield losses in wheat were due to pathogens (Oerke, 2006). *Fusarium* head blight (FHB) causes severe damages on wheat and other small grain cereals (Zhu et al., 2012). Although several fungal species of the *Fusarium* genus can cause FHB, the ascomycete fungus *F. graminearum* (telomorph: *Gibberella zeae*) is a major causal agent for FHB (Jung et al., 2013). Fg parasitizes roots, stems, leaves, and reproductive tissues of many species of cereals and grasses. FHB potentially causes dramatic economic losses in wheat, barley and other grasses (Bischof et al., 2011; Montibus et al., 2013). In cereals grain, FHB results in accumulation of trichothecene mycotoxins which are harmful for humans and passes to animals (Desjardins and Hohn, 1997; Jia et al., 2009).

Plants usually cope with different environmental changes such as cold, salt, or drought stresses and biotic stresses that are induced by microorganisms or herbivores in their habitats. One of the major biotic threats for plant survival is exposure to pathogens. Therefore, plants have evolved complex strategies to allow rapid modulation of cellular functions of an active defense response (Li et al., 2006). First of all, for a successful defense response, plants must rapidly recognize the environmental changes and respond to them accordingly. In plants, induced defense responses are activated by the perception of pathogen-associated molecular patterns (PAMPs) found on the pathogen surface (Asai et al., 2002; Boller and He, 2009). PAMP-triggered immunity (PTI) activates not only the mitogen-associated protein kinase (MAPK) cascade but also the *WRKY* transcription factors (TFs) which are the members of PTI and regulate basal resistance (Asai et al., 2002; Shen et al., 2007).

The pathogen responsive gene expression plays a crucial role in plant responses. One of the ways to regulate transcription is to utilize the function of TFs. To date, various TFs have been identified in plants. Among TFs, the basic leucine zipper protein (bZIP) family was reported to have various biological functions in plants such as development processes and stress responses (Zhang et al., 2008; Baloglu et al., 2014a; Hwang et al., 2014). In addition, WRKY TF family is also found in the plant kingdom and contains different WRKY families in higher plants (Ulker and Somssich, 2004). They play crucial roles in the regulation of many plant biological processes including the pathogen infection (Eulgem and Somssich, 2007).

Microarray platforms for transcriptome analysis are useful tools for functional genomics since they can provide the observation of gene expression profiles systematically. In plants, these analyses have been applied for stress response or developmental studies that can provide new insights to understand the pathogen-host interaction in different tissues, time points, and pathogens. Thus, it can be useful to use a time course combined with alternate plant genotypes (Wise et al., 2007).

Despite the agronomical importance, knowledge on mechanisms of pathogen resistance in *Triticeae* is limited (Bischof et al., 2011). In this respect, the control of FHB is known to be difficult because the lack of host-pathogen interaction information at the molecular level (Yang et al., 2010). To date, different studies have been conducted to find out wheat responses to pathogenic stress at the molecular level. Plant—Fg interaction studies were performed to investigate the response of susceptible, moderately susceptible, and resistant wheat cultivars in different tissues (glume, lemma, palea, anther, ovary, and rachis) and time points by using microarrays (Bernardo et al., 2007; Golkari et al., 2007) and cDNA-AFLPs methods (Steiner et al., 2009). Jia et al. (2009) examined disease severity, deoxynivalenol (DON), fungal biomass, and transcript accumulation in a wheat near-isogenic line pair carrying either the resistant or susceptible allele for the chromosome 3BS FHB-resistance quantitative trait locus (*Fhb1*). According to the their results, wheat exhibits both induction and repression of large sets of gene transcripts during *F. graminearum* infection. In addition, a difference in the general timing of transcript accumulation in plants carrying either the resistant or susceptible allele at the *Fhb1* locus was detected, and 14 wheat gene transcripts were detected that exhibited accumulation differences between the resistant and susceptible alleles (Jia et al., 2009). Genome-wide analysis was conducted to study *Blumeria graminis* f. sp. tritici (Bgt) infection in susceptible and resistant wheat in response to powdery mildew infection (Xin et al., 2012). In order to analyze further elucidation of wheat defensive-response to powdery mildew, a gene-chip was used (Wang et al., 2012). However, no experiment has been designed to detect the early response mechanism of susceptible and moderately susceptible wheat cultivars by using a chip that contains 12X135K unigenes in wheat genome wide array. To achieve this, the gene expression levels of two genotypes were compared after being infected with Fg. To identify the early response of wheat cultivars at seedling stage to pathogenic Fg, overall view of the differences between the infected susceptible and moderately susceptible winter wheat cultivars, and also between the before and after infection of each cultivar was demonstrated in this study by comparing the gene expression levels. The differentially regulated genes in response to pathogenic stress caused by Fg were functionally classified by using Gene Ontology (GO) and KEGG analysis.

## Materials and methods

### Plant materials, growth conditions, and inoculation

Two winter wheat cultivars were used in the study; moderately susceptible (MS) cv. “Mizrak 98” and susceptible (S) cv. “Gun 91” to *Fusarium* head blight (FHB) (Demirci, 2003). Pedigree and cultivar information of the cultivars can be found at www.wheatatlas.org. The seeds were surface sterilized by soaking in 2% sodium hypochlorite for 20 min and than rinsed three times with sterile water. The sterilized seeds were sown in a mixture of sterile peat, sand, and soil in a ratio of 1:1:1. Plants were grown in a controlled environment in a climate cabinet for 8 days under 24°C 16/8 h light/dark.

*Fusarium* isolate H10Z32-B2 (kindly provided by Prof. Dr. Berna Tunali from the Department of Plant Protection, Faculty of Agriculture, 19 Mayis University) was grown in potato dextrose agar (PDA) medium. The root crown of each plant was inoculated with injection at the seedling stage with a freshly prepared conidial suspension (1 × 10^5^ spores/ml). Inoculated plants were incubated for 12 h in a growth cabinet under 100% relative humidity (Inal et al., 2014). Control plants of the same line were injected with distilled water and incubated under the same conditions. The two uppermost fresh leaves of mock and pathogen inoculated plants were harvested 0 and 12 h after inoculation and stored at −80°C until processed.

### RNA isolation, labeling, and hybridization

Two biological replicates were used for each cultivar and their controls in the analyses. Frozen leaf tissues were ground to a fine powder in liquid nitrogen. Total RNA extraction was performed with TRIzol® Reagent (Invitrogen, Cat No. 15596-026) in line with the manufacturer's protocol. To remove the DNA contamination in samples, DNase I (Fermentas, Vilnius, Lithuania) was used according to the manufacturer's instructions. The quality and quantification of total RNA was assessed by NanoDrop 2000c spectrophotometer (Thermo Fisher Scientific, Lenexa, KS, USA), and RNA quality was checked on 1.5% agarose gel.

cDNA was generated from 10 μg of total RNA using the SuperScript Double-Stranded cDNA Synthesis Kit (Invitrogen, Carlsbad, CA, USA), and labeling with Cy3 random nanomers was performed using One-Color DNA Labeling Kit (Roche NimbleGen, Inc. Madison, WI, USA) following the manufacturer's instructions. Four microgram labeled products were loaded onto the custom 12X135K array, incubated at 42°C over night in a Hybridization System, and washed in the NimbleGen Wash Buffer Kit following the NimbleGen protocol. Microarray slide, scanned with 2 μm resolution using a MS 200 Microarray Scanner, generating the corresponding 532 nm TIFF images was used. Then, the data were imported into the DEVA software to quantify the signal intensities of the spots on the image (Turktas et al., 2013).

### Microarray design and data analysis

Experiments were repeated with two independent biological replicates. A newly developed large-scale custom INRA GDEC *Triticum aestivum* NimbleGen 12X135K unigenes microarray including 39,179 oligonucleotide probes was used for the representation of the wheat transcriptome. The probes were spotted as duplicates and triplicates on the array.

Transcriptome profiling was detected over a time course for both the infected and control plants collected at 0 and 12 hai. The array data was normalized according to the quantile method for standardization (Bolstad et al., 2003) and the Robust Multichip Average (RMA) algorithm (Irizarry et al., 2003) with DEVA software. The signal intensities of the samples were transformed into log2-ratio data. The dye-normalized and background-subtracted intensity data were exported into the ArrayStar software (DNAStar, Madison, WI, USA) to perform gene expression analyses. The Student's *t*-test was used to identify differentially expressed genes. *P* ≤ 0.05 and 1.5-fold change were used to consider differential expression of genes between the two data sets.

### Data mining

The Basic Local Alignment Search Tool (BLAST) algorithm and BLAST to Gene Ontology (Blast2GO) tool were used and compared with the National Center for Biotechnology Information (NCBI) database to annotate the genes corresponding to the hybridized cDNA signals. Gene ontology analysis was performed using the Gramene (http://www.gramene.org) ontology tool. The MapMan software tool (Usadel et al., 2005) was used to display differentially expressed genes in response to pathogenic stress. MapMan mapping file and bincodes for *Triticum aestivum* L. were extracted from dbWFA, a web-based database for the functional annotation of wheat (Vincent et al., 2013). To elucidate the metabolic pathways of these predicted genes, Blast2GO was applied to assign Kyoto Encyclopedia of Genes and Genomes (KEGG) pathways of differentially expressed genes.

### qRT-PCR

The expression profiles of the randomly selected seven genes were measured via quantitative PCR reactions (qRT-PCR) in order to confirm the microarray data. The sequences of gene specific PCR primers used in qRT-PCR have been listed in Table 1. The primers were designed using Primer3Plus software version 2.3.3 (http://primer3plus.com) (Untergasser et al., 2012).

**Table 1:** **Selected genes and their primer sequences used for the validation of microarray results**.

**SEQ ID**	**Description**	**Forward Primer (5′->3′)**	**Reverse Primer (5′-3′)**
Ta_S37854776	Peroxidase 12	CACTCCGAACATCGACTTCA	CCCTGGTCTGACTTGAACAG
Ta_S46915595	Gamma-gliadin	CATACAGATCCTGCGACGAC	GCAGTCAGGTCGGACATACA
Ta_S13166630	Unknown	TGCTCACCCAGCAGAGTTAC	TGTCTGGAAAGTGGACCATTG
Ta_S12917674	Mitogen-activated protein kinase kinase 6	GCATTGTGATCTGAAGCCTGTC	CCAGTTCCGGGAGCAGTTTA
Ta_S12983212	Glutathione transferase	CTTCACCGCCTGGTTCC	CAGACGTACGCATTGTAGGC
Ta_S17893086	Disease resistance response protein 206	GGAGGAGCTGTTGGTGGA	GCCCAGCAGCAAGACATT
Ta_S13176655	Probable wrky transcription factor 21-like	GGCCGATATACCTTCCGACA	CTCGTAGGTGACGGATGAGC

First strand cDNA was synthesized from total RNA that used in the microarray analysis using Fermentas First Strand cDNA Synthesis Kit (Thermo Fisher Scientific) according to the manufacturer's instructions. The qRT-PCR experiments were performed as previously reported by Turktas et al. (2013). cDNA (2 μl) was amplified with 0.1 μl specific primers in a total volume of 18 μl, using a LightCycler 480 Real-Time PCR System with SYBR Green I Master (Roche Applied Science, Penzberg, Germany). The 18S rRNA (GenBank ID: AF147501) was used as internal control. For each gene and 18S rRNA, standard curves were produced based upon serial dilutions of cDNAs, which were reverse-transcribed from target genes that were expressed at an appropriate level. The qRT-PCR reaction conditions were as follows: preheating at 95°C for 5 min; and 50 cycles of 95°C for 10 s, 53–55°C (depending on the primers' annealing temperature) for 20 s, and 72°C for 10 s. Three replicates were performed for each sample. The gene expression levels were calculated according to the 2^dΔCt^ algorithm (Schmittgen and Livak, 2008).

## Results

We examined the expression profiles of the genes in response to FHB in two wheat cultivars for five time comparisons (Gun-91 12 hai/Gun-91-12 hai control (C), Gun-91 12 hai/Gun-91 0 hai, Mizrak 12 hai/Mizrak C, Mizrak 12 hai/Mizrak 0 hai, and Mizrak 12 hai/Gun-91 12 hai) using Nimblegen 12X135K expression array. Two biological replicates were used for this analysis.

### Microarray analyses

**Figure 1:**
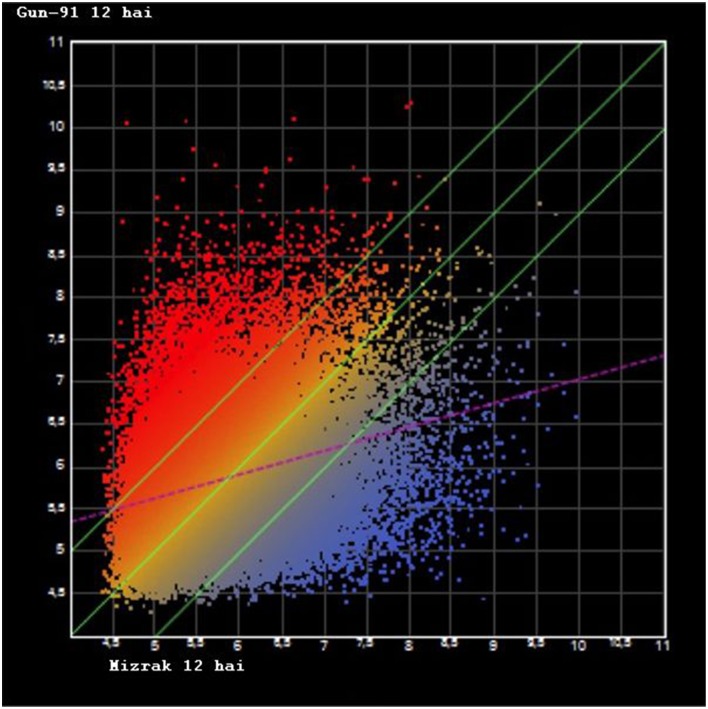
**Scatter plot graph of differentially expressed genes after microarray hybridization**. X axis represents the Gun-91 12 hai, Y axis represents the Mizrak 12 hai (hai: hours after inoculation).

**Figure 2:**
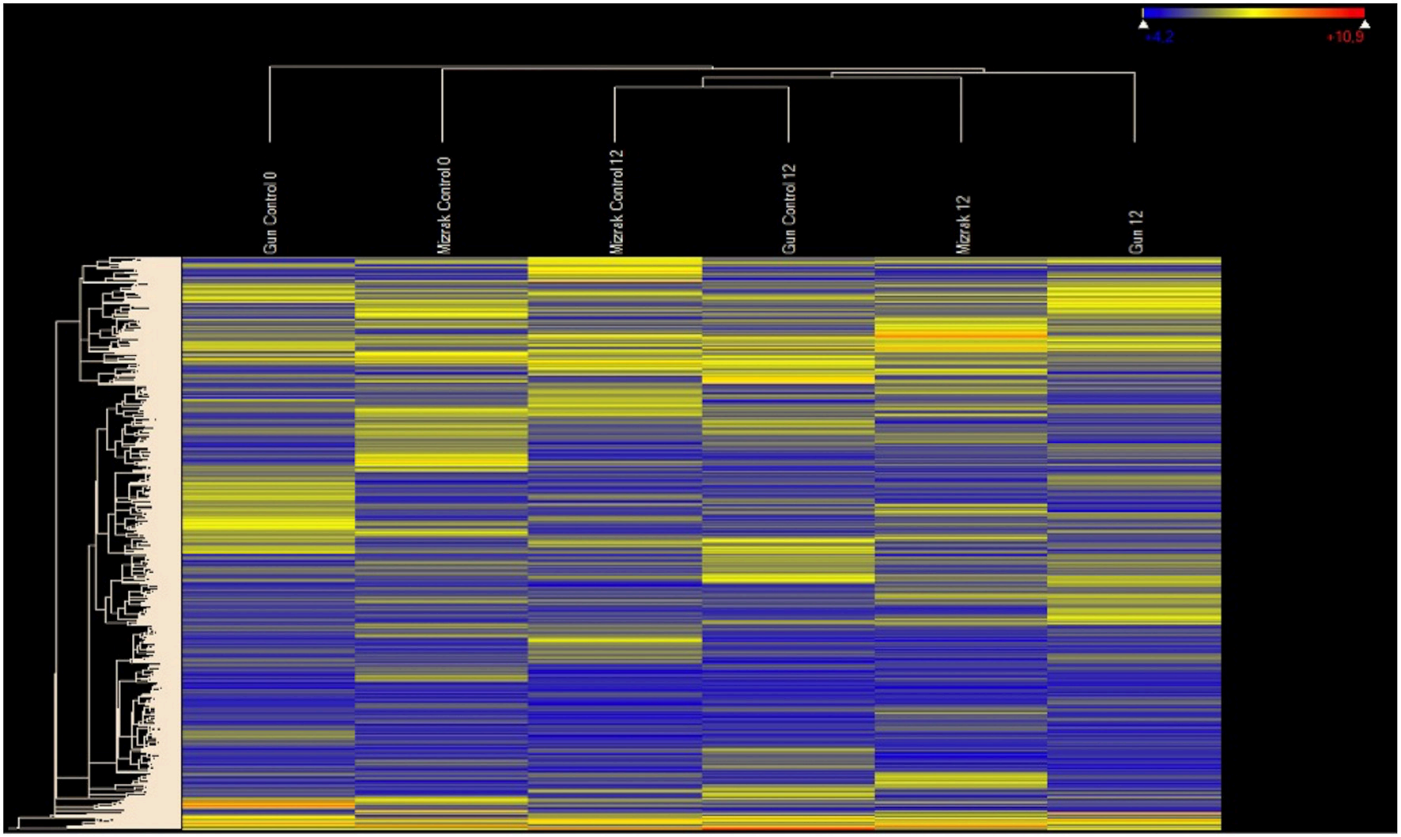
**Heat map illustrating the hierarchical clustering results of microarray expression profile data**. As a global observation, this heat map indicates differential regulation signatures in response to Fusarium inoculation for different time points. MR cultivar Gun is distinctly separated from others.

NimbleGen gene expression array was used to examine the transcription profiling of the two cultivars at two different time points. A total of 39,179 oligonucleotide probes were used for the microarray analysis and all the probes produced detectible signals (Supplementary Table 1). To investigate the variation of gene expression levels between the two data sets, scatter plots were generated. In Mizrak 12 hai vs Gun-91 12 hai, *R*^2^-value was found as 0.08, Mizrak 12 hai vs. its control *R*^2^ were 0.09 and Gun 12 hai vs its control it was 0.07 (Figure 1). Therefore, Pearson correlation coefficients indicated that expression levels between comparisons were mostly identical. A heat map, constructed using the result of microarray analysis, demonstrated that there were two main generated groups (Figure 2).

### Validation of differential expression via qRT-PCR

**Figure 3:**
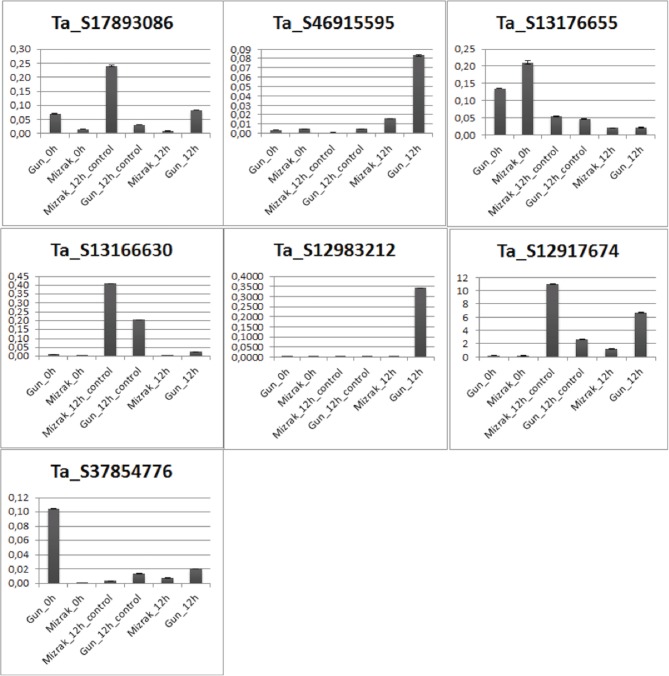
**Verification of microarray results by real-time quantitative RTPCR**.

Expression levels of differentially regulated genes was validated using qRT-PCR on samples of both Gun-91 (S) and Mizrak (MS) collected at 12 hai following Fg inoculation. The results of qRT-PCR showed an almost similar pattern with those obtained from array data, with some exceptions. Among the seven selected genes, qRT-PCR expression profiles of six genes, *mitogen-activated protein kinase kinase 6* (Ta_S12917674), *disease resistance response protein 206* (Ta_S17893086), *probable wrky TF 21-like* (Ta_S13176655), *peroxidase 12* (Ta_S37854776), *gamma-gliadin* (Ta_S46915595), and an unknown protein (Ta_S13166630), showed positive correlation with the microarray data (Figure 3). For instance, *mitogen-activated protein kinase kinase 6* was detected as up-regulated in the microarray analysis with a fold change of 3.1 in Mizrak at 12 hai compared to that of Mizrak at 0 hai, while in qRT-PCR results 8-folds were observed. It could be due to the sensitivity of the qRT-PCR. The transcript encoding *peroxidase* 12 was also in corroboration with the array data in Mizrak 12 hai vs its control with a fold change of approximately 2. On the contrary, *glutathione transferase* (Ta_S12983212) did not show any similarities in the microarray data in which the gene was detected as down-regulated in susceptible cultivar at 12 hai, while in qRT-PCR, it was detected as up-regulated. In general, most of the transcripts analyzed with qRT-PCR showed correlation with the microarray data (Figure 3).

### Comparative analysis of transcriptional profiles

**Figure 4:**
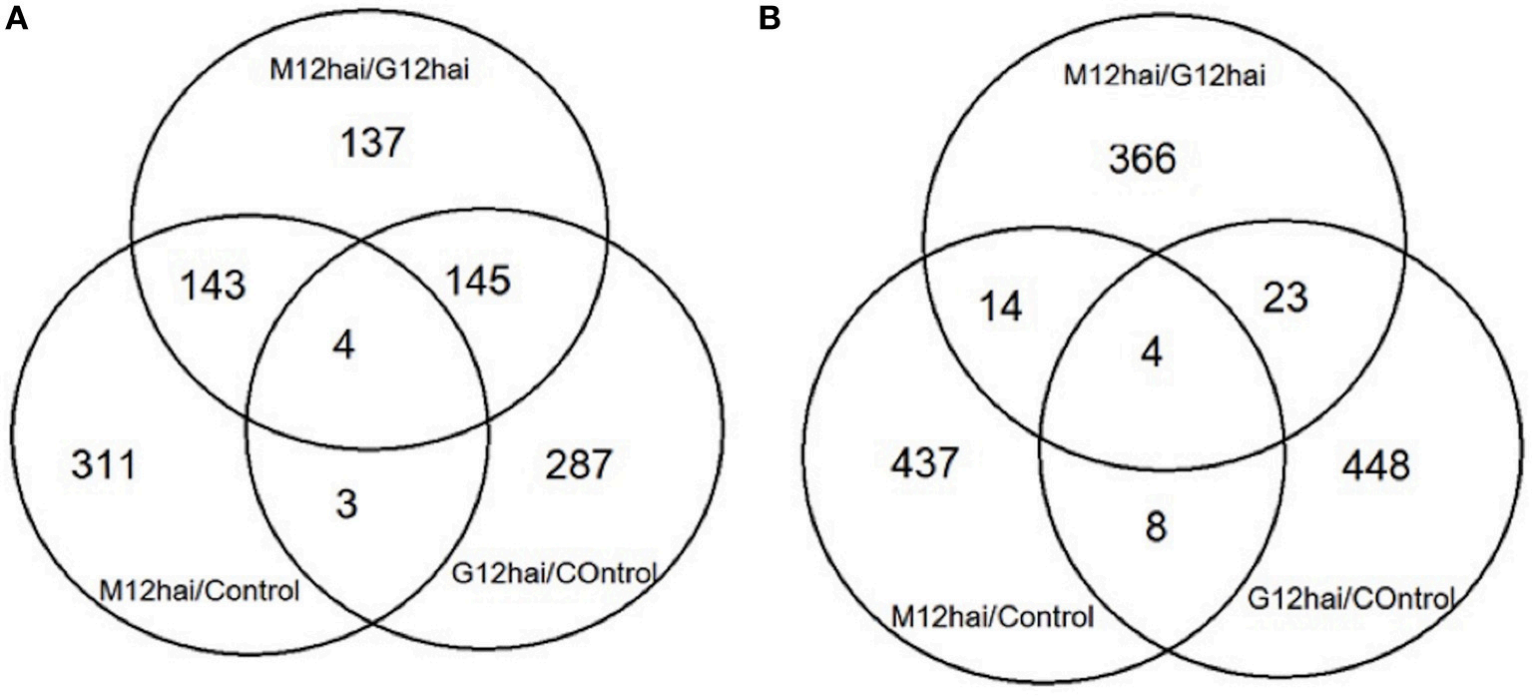
**Venn diagrams displaying the differentially expressed overlapped and unique genes upon Fusarium inoculation after 12 h. (A)** Displaying the number of up-regulated genes **(B)** displaying the number of down-regulated genes. Significance was set at *P* < 0.05, with a fold-change of 1.5 (log2 scale). M12hai = Mizrak 12 h after inoculation, G12hai = Gun-91 12 h after inoculation.

Differentially expressed transcripts were evaluated according to the *t*-test (*p* ≤ 0.05 and 1.5-fold) and listed in Supplementary Table 2. The results revealed that 3668 genes, which account for 9.4% of the probes in the microarray, were significantly differentially expressed at least in one time comparison. Additionally, there were 29 transcripts that showed > ±4-fold change differentiation between the libraries (Table 2). Venn diagrams (Figure 4) represent the number of up- or down-regulated transcripts among comparisons upon pathogen infection and also display the overlapping transcripts as well as the unique ones for each condition. Although the number of altered transcripts was almost the same for all comparisons, the data demonstrated that the number of repressed unique genes was higher than the up-regulated ones.

**Table 2:** **Differentially expressed genes showing ±4-fold change at least one time course**.

	**Description**	**G12/C**	**G12/G0**	**M12/C**	**M12/M0**	**M12/G12**	**GO biological process**
Ta_S12902838	Hypothetical protein	↔	↑	↔	↔	↔	Unknown
Ta_S12983212	Glutathione transferase	↔	↔	↔	↔	↑	Response to oxidative stress, response to cold, response to herbicide, glutathione metabolic process
Ta_S13020758	Unknown	↔	↔	↔	↑	↔	Unknown
Ta_S13112037	ap2 erebp transcription factor superfamily protein	↔	↓	↔	↔	↔	Response to salt stress, regulation of transcription, organ morphogenesis, vegetative to reproductive phase transition of meristem, response to abscisic acid, transcription stimulus
Ta_S13122648	Hypothetical protein TRIUR3_27413	↓	↔	↔	↔	↔	Unknown
Ta_S13134419	Hypothetical protein F775_22156	↑	↑	↔	↔	↔	Unknown
Ta_S13144312	Alternative oxidase	↔	↑	↔	↑	↔	Generation of precursor metabolites and energy, oxidation-reduction process, oxidoreductase activity
Ta_S13166630	Unknown	↑	↔	↔	↔	↓	Unknown
Ta_S13185742	Unknown	↔	↔	↔	↔	↑	Unknown
Ta_S13189037	Unknown	↔	↑	↔	↔	↔	Unknown
Ta_S16205726	Coiled-coil domain-containing protein 65	↓	↔	↔	↔	↔	Unknown
Ta_S16256800	DNA topoisomerase expressed	↔	↑	↔	↔	↔	Unannotated
Ta_S17893086	Disease resistance response protein 206	↑	↑	↔	↔	↔	Unannotated
Ta_S17893772	Cyclin-dependent kinase c-2-like	↔	↔	↑	↑	↔	Response to virus, protein phosphorylation, carpel development, regulation of viral process, leaf development, mRNA processing
Ta_S17896054	Phospholipid-transporting atpase 2-like	↔	↔	↔	↑	↔	Phospholipid transport, phospholipid-translocating ATPase activity, cytoplasmic membrane-bounded vesicle
Ta_S17977116	Hypothetical protein F775_05471	↔	↔	↔	↑	↔	Unannotated
Ta_S17987494	f-box protein pp2-a13	↑	↔	↔	↔	↓	Unannotated
Ta_S22369341	Glycosyltransferase	↓	↔	↔	↔	↔	Transferase activity
Ta_S24623090	Allene oxide cyclase chloroplastic	↔	↔	↔	↑	↔	Allene oxidase cyclase activity
Ta_S26021235	Unknown	↑	↑	↔	↔	↔	Unknown
Ta_S26026058	Ferredoxin- chloroplastic-like	↔	↑	↔	↔	↔	Electron transport chain, zinc ion binding, Chloroplast
Ta_S32494292	Polygalacturonase procursor	↓	↔	↔	↔	↔	Unannotated
Ta_S32531082	Auxin responsive factor 16-like	↔	↔	↓	↔	↔	Unknown
Ta_S32599956	Protein	↔	↓	↔	↔	↔	Single-organism cellular process, single-organism transport, membrane
Ta_S32694279	Methionyl-trna formyltransferase-like	↔	↔	↓	↔	↔	Translational initiation, conversion of methionyl-tRNA to N-formyl-methionyl-tRNA, purine ribonucleotide biosynthetic process
Ta_S37852892	Unknown	↔	↔	↓	↔	↔	Unknown
Ta_S37887180	Unknown	↔	↔	↔	↔	↓	Unknown
Ta_S38676400	Alpha-gliadin	↓	↔	↔	↔	↔	Nutrient reservoir activity
Ta_S50084340	Unknown	↔	↔	↔	↔	↑	Unknown

*The symbols indicate (↑) up-regulation (↓) down-regulation (↔) no significant change*.

According to the Venn diagram (Figure 4), the comparison of three groups showed that they shared 8 transcripts in common. Annotation of these common transcripts revealed that they were mainly involved in response to stimuli, metabolism, and gene expression processes.

### Classification of differentially expressed gene response to FHB

**Figure 5:**
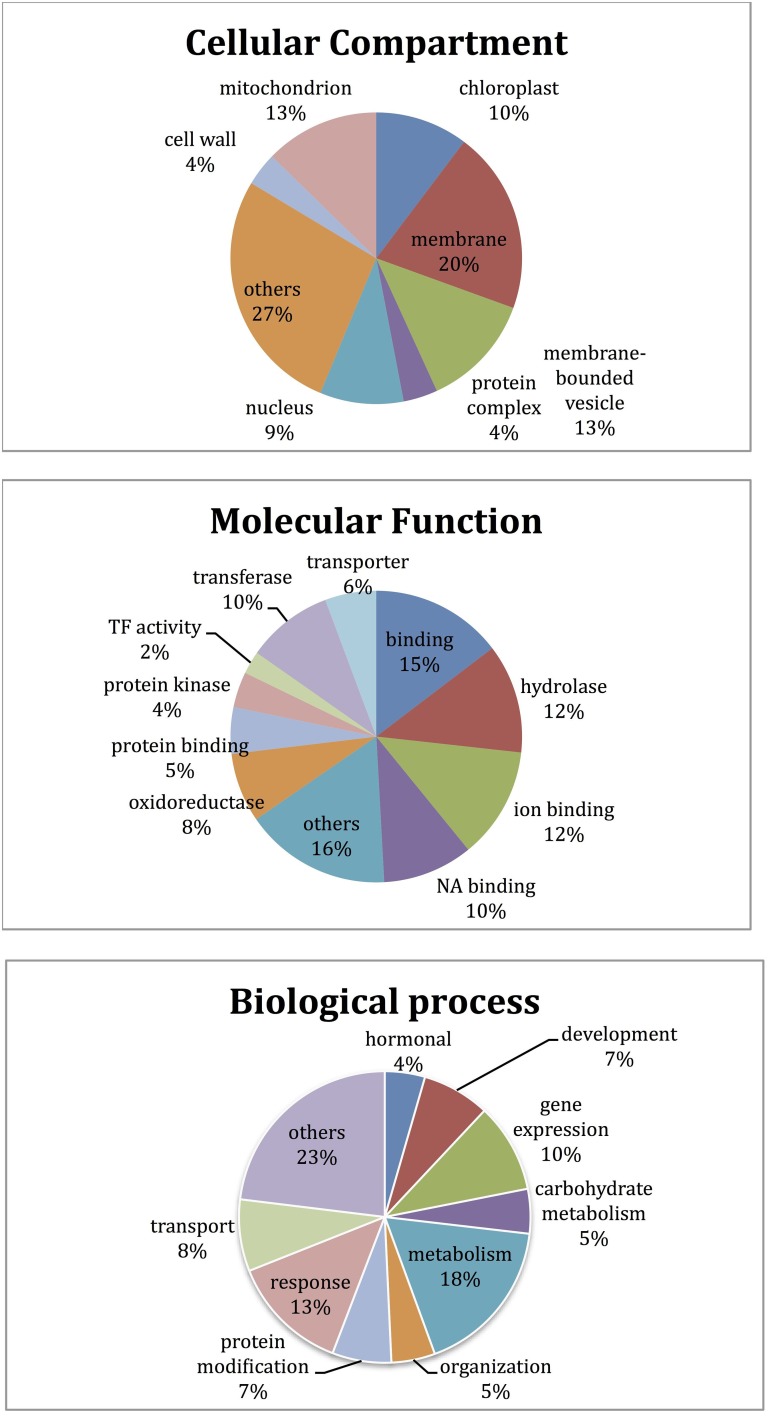
**Functional GO categories' distribution of differentially expressed transcripts**.

GO enrichment analysis was performed for the classification of differentially expressed genes (Supplementary Table 3). Among those genes, 1419 (39%) had known functions, while the majority had unknown and/or unclassified functions. Among the most abundant transcripts involved in diverse biological processes, most belonged to metabolic process, response to stimuli, and gene expression, respectively. The transcripts playing role in membrane and membrane-bounded vesicles and binding were represented with a high ratio as well (Figure 5).

**Figure 6:**
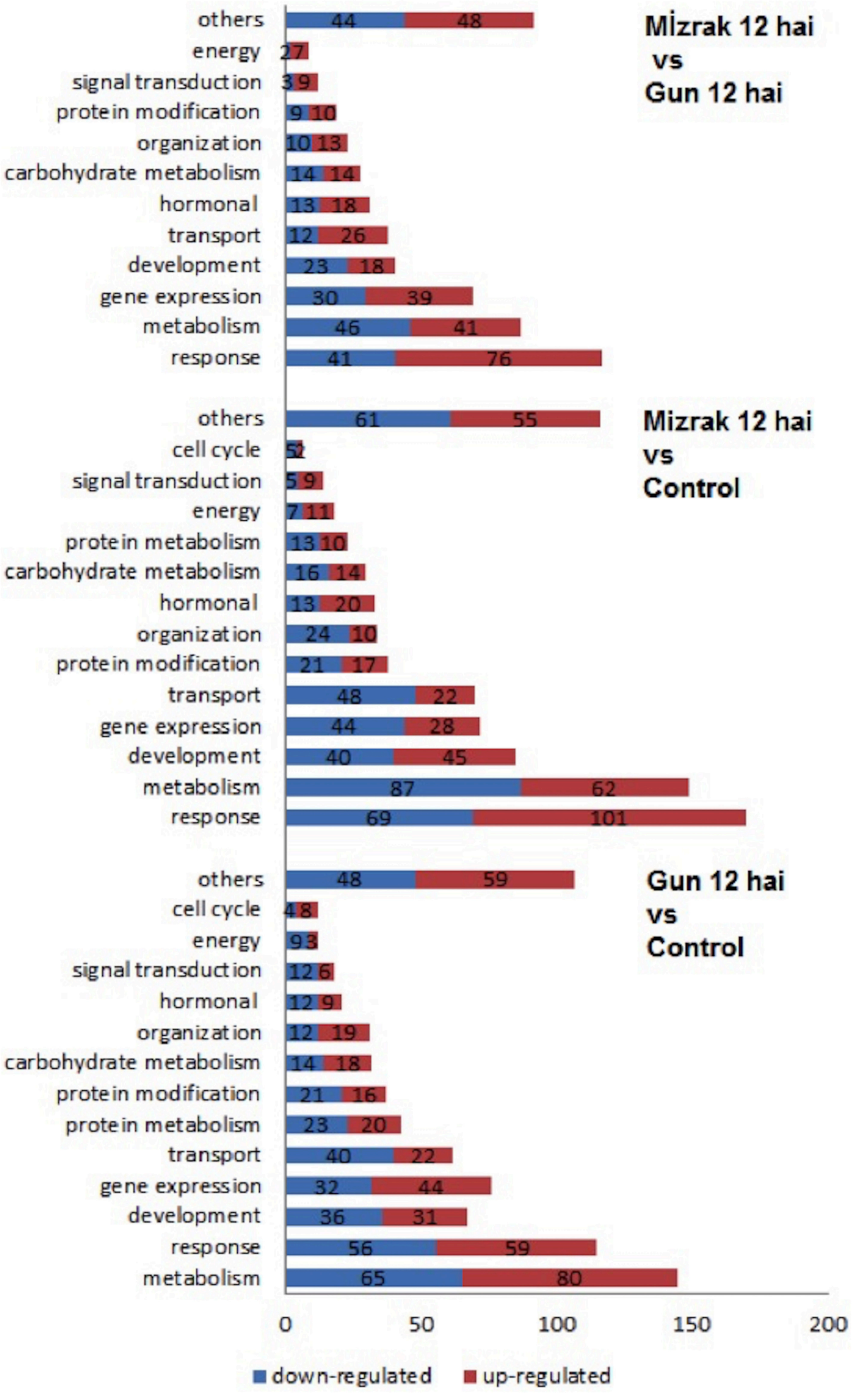
**Bar graph representation of gene ontology for genes differentially expressed in microarray analyses according to the biological process**. Gene expression alterations in wheat leaves were compared to their controls and within each other.

Microarray results showed that 922 transcripts in cv. Gun 91 (S) were differentially expressed in infected samples compared to the control group. Functional classification of differentially expressed genes using GO analysis revealed that 778, 555, and 562 GO terms were involved in biological process (GO:0008150), molecular function (GO:0003674), and cellular compartment (GO:0005575), respectively. The results indicated that the largest group of transcripts belongs to metabolic process (GO:0008152) classified within biological process (Figure 6). Under metabolic process, the majority of the transcripts associated with energy process, transport, and signal transduction were down-regulated in infected samples. Considering the cell compartmentalization, the highest number of transcripts was found to be membrane associated transcripts.

When Mizrak (MS) at 12 hai was compared to its control, 924 transcripts were differentially expressed. The GO annotation analysis of the differentially expressed transcripts revealed that 860, 545, and 502 GO terms were related to biological process, molecular function, and cellular compartment, respectively. Annotated transcripts, belonging to response to stimuli and stress (GO:0050896), metabolic processes (GO:0008152), and development (GO:0032502) were significantly affected by pathogenic stress (Figure 6). A large number of genes related to response to stimuli was up-regulated, while the number of down-regulated genes was higher in metabolic process and gene regulation. Further, the transcripts belonging to gene expression and transport showed significant alterations. In the process of response to stimuli, transcript accumulation was higher than that of Mizrak than Gun-91. Additionally, in metabolism the abundant number of transcripts was negatively expressed whereas energy related transcripts were positively regulated.

We found that 835 transcripts were significantly regulated in infected MS cv. “Mizrak” compared to infected susceptible cv. “Gun 91.” The GO annotation analysis of the differentially regulated transcripts showed that there were 555, 408, and 363 GO terms involved in biological process, molecular function, and cellular compartment, respectively. As shown in Figure 6 and Supplementary Table 3, the transcripts related to response to stimuli, metabolic process, and gene expression were significantly affected by pathogenic stress. At 12 hai, the number of up-regulated transcripts related to stress response and gene expression was higher in Mizrak (MS) than those of Gun 91 (S). This result indicates that MS cultivar has already been responsive to stress. In Mizrak, the transcripts involved in signal transduction, transport and membrane were found to be positively regulated with the ratio of 73, 68, and 61%, respectively. However, the number of down-regulated transcripts belonging to metabolic and developmental processes was higher. The transcripts related to protein complex showed a significant differentiation in gene accumulation. About 86% of the transcripts were highly expressed in Gun-91 such as *swı snf complex subunit swı3b-like* (Ta_S17989822), *eukaryotic translation initiation factor 3 subunit d* (Ta_S17890113), and *dna helicase ino-80-like* (Ta_S26028907).

### The transcripts involved in response to stimuli were the most affected by FHB

**Figure 7:**
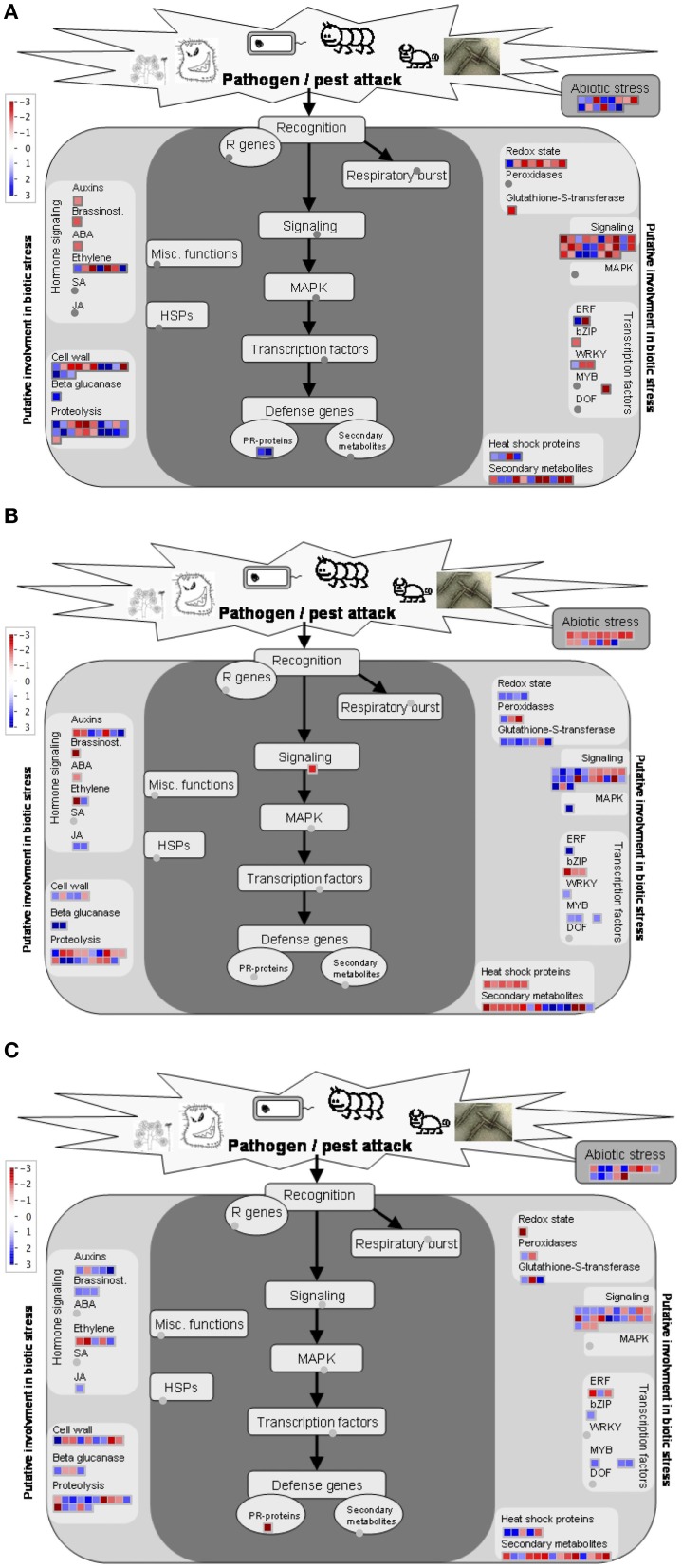
**MapMan overview showing differentially expressed genes related to biotic stress and stress response**. Colored boxes indicate the differentially expressed transcripts. Differentially expressed transcripts related to biotic stress and stress response in susceptible cultivar, Gun-91 **(A)** and moderately susceptible cultivar, Mizrak **(B)**, and comparison of cultivars **(C)** are displayed.

In all comparisons, transcripts involved in response to stimuli were among the most abundant ones (Figure 6). Differentially expressed genes more specifically related to biotic stress were displayed using MapMan software tool onto biotic stress and stress response overview. It should be noted that the MapMan mapping file cannot cover all differentially expressed transcripts determined via microarray analysis. As shown in Figure 7A, the strongly regulated biotic stress related genes in Gun 91 (S) compared to its control include two PR-proteins [chitinase A (Ta_S13112328), disease resistance—responsive protein (Ta_S17893086)], several proteinase inhibitors, β-glucanase (Ta_S32704565), glutathione-S-transferase (Ta_S37823230) (Figure 7A). The transcripts Ta_S17989500, Ta_S16058219, and Ta_S38675474 related to auxin-responsive protein, brassinosteroid, and ABA signal transduction, respectively, were all down-regulated in Gun 91 (S). On the other hand, seven transcripts involved in ethylene signaling and biosynthesis were differentially regulated. It was also found that seven TF-related genes were differentially regulated in Gun 91 (S). In MS cultivar, Mizrak, the significantly regulated biotic stress related genes compared to its control include a 1,3-β-gluconase (Ta_S13000333), three peroxidases (Ta_S18006573, Ta_S18011877, Ta_S37760603), seven Glutathione-S-transferases (Ta_S13048864, Ta_S13109610, Ta_S13111648, Ta_S17892747, Ta_S18009609, Ta_S18011877, Ta_S32697004), and several proteinase inhibitors (Figure 7B). A number of auxin-responsive genes were differentially expressed and two jasmonic acid related genes (Ta_S12923155 and Ta_S24623090) were down-regulated in Mizrak (MS) in response to FHB. When we compared the differentially regulated genes in Gun 91 (S) and Mizrak (MS), the responses of susceptible and MS cultivars to pathogen were different from each other with regards to gene expression regulation. For instance, we observed two defense genes (PR proteins) differentially regulated in Gun 91 (S), but not in Mizrak (MS). On the other hand, three peroxidases and two jasmonic acid related genes were differentially regulated in Mizrak (MS), but not in Gun 91 (S). Hence, it can be argued that both susceptible and MS cultivars were affected by the pathogen during 12 h of infection, but their responses to pathogen were quite different.

Comparison of the expression level of biotic stress related genes expressed in infected MS cultivar, Mizrak, and in infected susceptible cultivar, Gun 91, revealed that a number of biotic stress related genes differentially expressed include a PR-protein (Ta_S17893086), three β-gluconase (Ta_S26022505, Ta_S32554879, and Ta_S33578944), two peroxidases (Ta_S 17987935 and Ta_S37760603), three glutathione-S-transferase (Ta_S17892747, Ta_S26018841, and Ta_S37875911), and several proteinase inhibitors (Figure 7C). It is observed that the genes related to brassinostroid and jasmonic acid signaling pathway were up-regulated in Mizrak (MS) compared to those in Gun 91 (S). Of the five transcripts related to auxin-responsive proteins, four were up-regulated and the other one was down-regulated in Mizrak (MS).

The differentially regulated transcripts in each comparison were also subjected to annotation by using the Blast2GO software tool to determine their functions in a more detailed manner. In this study, PR genes such as peroxidase, chitinase, and endo-β-glucanase encoding transcripts were investigated as altered PR transcripts. Chitin, a substrate of chitinase, is the fundamental component of fungal cell wall, and the expression of many chitinase genes are induced by fungal pathogens (Zhong et al., 2002). Chitinase enzymes are known to have a role in chitin catabolism and plant defense. The data obtained from microarray analysis indicated that *chitinase 8* (Ta_S26026095) transcripts showed up-regulation pattern in Gun-91 at 12 hai compared to that of Mizrak. Moreover, *chitinase 8* (Ta_S26026095; Ta_S15880319) was detected as up-regulated in Gun 91 when compared to its control. In addition to those transcripts, *Chitinase-like protein 1-like* transcripts were up-regulated in Gun-91, whereas *chitinase 8* (Ta_S26026095) and *chitinase-like protein 1-like* (Ta_S26027709) were down-regulated in Mizrak 12 hai.

Endo-β-glucanase transcripts were found to be differentially regulated in this study. In Mizrak at 12 hai, *endo-*β*-glucanases* encoded by Ta_S13000333 and Ta_S33578941 were up-regulated 3.1- and 1.6-fold, respectively. On the other hand, the comparison of MS and susceptible cultivars indicated that the expression level of *endo-*β*-glucanase* (Ta_S33578944) in Gun-91 (S) was higher than that of Mizrak (MS). It was observed that the types of Peroxidase transcripts differed in Mizrak 12 hai compared to that of Gun-91 12 hai. In Mizrak, *peroxidase 15-like* (Ta_S12995082) and *peroxidase 1-like* (Ta_S17987935) genes were up-regulated, while *peroxidase 2* (Ta_S37760603) was down-regulated. ABC transporters, pleiotropic drug-resistant proteins and cytochrome p450 have been known to play a role in the detoxification process during fungal infection (Kosaka et al., 2015). In all time courses, the expressions of different ABC transporter family members were determined. Particularly, ABC transporter family members were found to be mostly induced by infection in Gun-91 (S). Pleiotropic drug-resistant proteins were also seen to be up regulation in Gun-91 (S) at 12 hai compared to Mizrak (MS) at 12 hai.

### bZIP and WRKY TFs are functional in regulating gene expression upon defense response

When TFs activity was analyzed, of the 57 transcripts in total, 48 transcripts were found to be differentially expressed at least one time comparison. In Gun-91 (S) at 12 hai compared to its mock control, there were 8 TFs that were regulated significantly. Of those, five of them were repressed, 3 of them were induced. The most repressed transcript was *wrky TF partial* (Ta_S44692914) with the fold change of 2.1, whereas the most up-regulated was *ap2 erebp TF superfamily protein*s (Ta_S32583333) with the fold change of 2.1. Thirteen transcripts corresponding to TF were detected in Gun-91 12 hai/G 0 hai time course. Of these differentially expressed genes, eight were up-regulated, while five were down-regulated. Furthermore, *AP2 EREBP TF superfamily protein* (Ta_S13112037) seems to be a significantly down-regulated gene with the fold change of 4.7.

Among the 15 transcripts differentially expressed in Mizrak (MS) at 12 hai, only five transcripts were up-regulated, whereas the remaining ten transcripts were down-regulated compared to its mock control. It was previously reported that bZIPs were the TFs in plants having role in pathogen defense (Zhang et al., 2008). In our study, *bzip TF superfamily protein* (Ta_S18007507) showed significant down-regulation pattern with the fold change of 3.2, while *wrky TF* 70 (Ta_S36162740) showed up-regulation pattern with the fold change of 3. In addition, *wrky TF* 70 (Ta_S36162740), which has a role in balancing salicylic acid (SA)- and jasmonic acid (JA)-dependent defense pathways detected as up-regulated in Mizrak (MS) when compared to Gun-91 (S).

Comparison of the differentially regulated transcripts in susceptible and MS cultivars revealed that a total of 10 transcripts were found to be common. Of these transcripts, five were up-regulated and the other five were down-regulated. *c-repeat binding factor 3-like protein* (Ta_S35718022) was the most up-regulated transcript (2.4-fold) in Gun-91 (S), while *wrky TF 70* (Ta_S36162740) was in Mizrak (MS). The transcripts taking part in gene expression were also identified in this study. The number of transcripts was the same in both cultivars but their regulations were opposite.

### Carbohydrate metabolism related transcripts' functions upon early defense response

**Figure 8:**
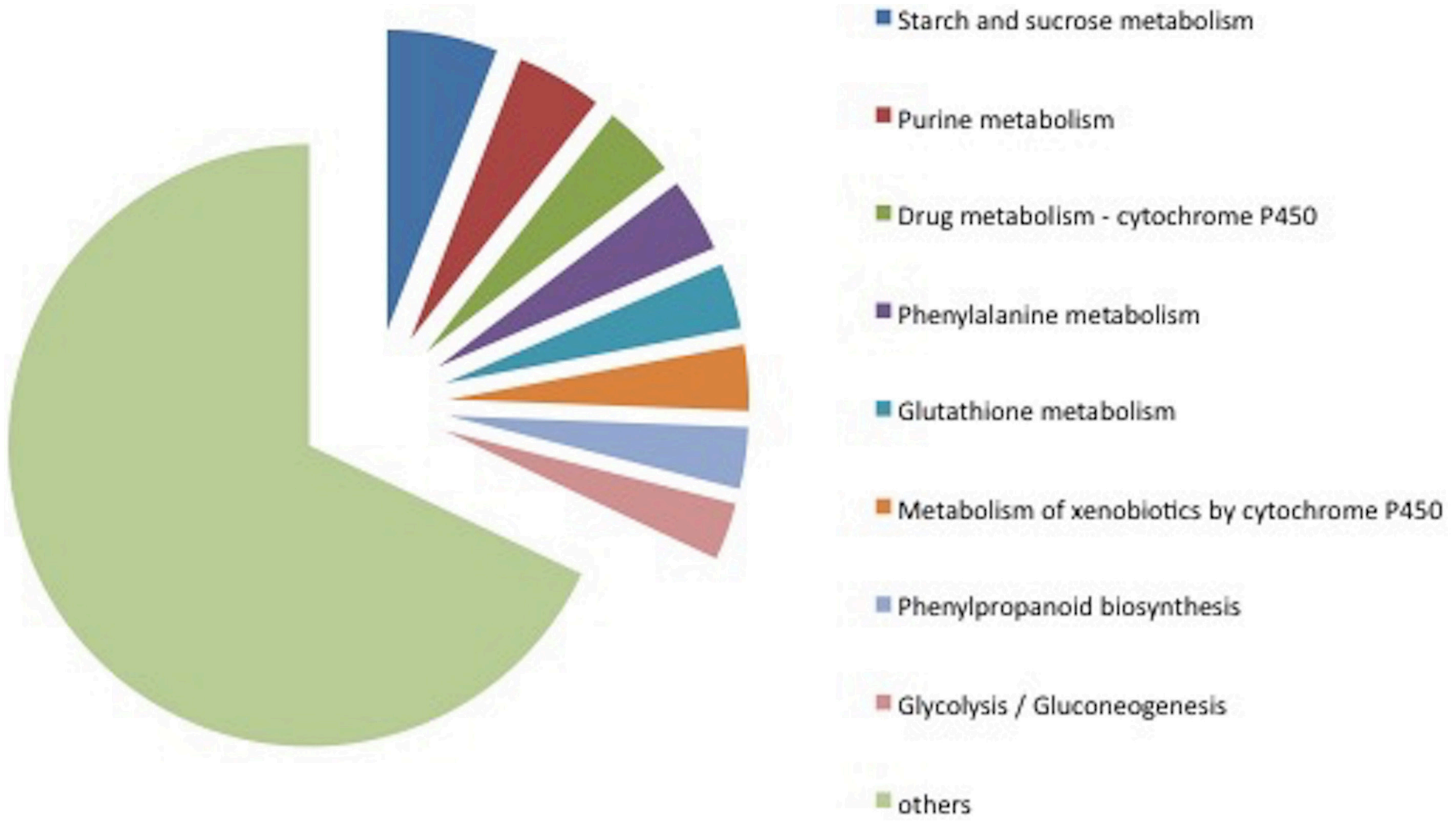
**Classification of KEGG pathways detected in microarray analysis**. The pathways displayed here include at least 14 transcripts.

**Figure 9:**
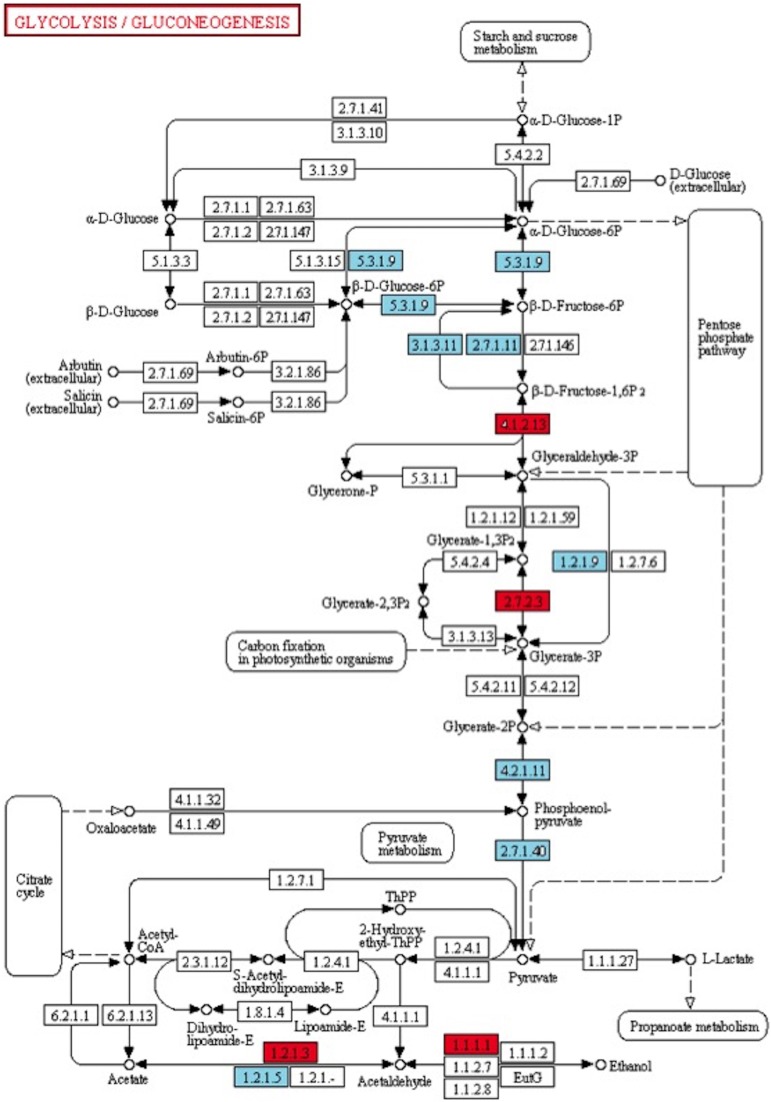
**KEGG pathway map analysis of Degs**. Glycolysis/Gluconeogenesis (map00010) is one of the significant pathway detected after Fusarium inoculation. Blue and red boxes indicate down- and up-regulation, respectively. The enzyme codes and corresponding transcripts are given below. ec:5.3.1.9: glucose-6-phosphate cytosolic (Ta_S32418945); ec:3.1.3.11: fructose-bisphosphatase, sedoheptulose—chloroplastic-like (Ta_S12922973, Ta_S26025373); ec:2.7.1.11: pyrophosphate-fructose 6-phosphate 1-phosphotransferase subunit alpha-like (Ta_S13144045); ec:4.1.2.13: fructose-bisphosphate chloroplast expressed (Ta_S17888674); ec:1.2.1.9: nadp-dependent glyceraldehyde-3-phosphate dehydrogenase (Ta_S13048872); ec:2.7.2.3: phosphoglycerate cytosolic-like (Ta_S32518390); ec:4.2.1.11: enolase chloroplastic-like (Ta_S17986798); ec:2.7.1.40: pyruvate kinase (Ta_S24623093); ec:1.2.1.3: aldehyde dehydrogenase (Ta_S13270649); ec:1.2.1.5: aldehyde dehydrogenase family 3 member f1-like (Ta_S12947041); ec:1.1.1.1: alcohol dehydrogenase 1 (Ta_S32643099).

Among the 285 transcripts found in the KEGG pathway analysis, 275 unigenes were classified into 74 pathways (Supplementary Table 4). The classification of the pathways was shown in Figure 8. In the KEGG pathways, the largest number of transcripts differentially expressed in response to *Fusarium* inoculation was involved in starch and sucrose metabolism and purine metabolism (Figure 8). Furthermore, a significant alteration was detected in the Glycolysis/Gluconeogenesis pathway in response to pathogenic stress. Through the KEGG analysis, we observed 28 altered transcripts in starch and sucrose metabolism and 14 transcripts in the Glycolysis/Gluconeogenesis pathway. The transcripts involved in these pathways were illustrated in Figure 9.

## Discussion

Large-scale gene expression analysis is mostly used to find out plant response to their environment (Desmond et al., 2008). Gene expression profiling upon biotic stresses has been broadly studied in different plant species using microarrays. Various stress treatments like pathogen stress have been investigated in different plants or in different tissues of plants. FHB is one of the most common diseases of wheat caused by Fg and, due to the limited molecular information on this disease, it is required to be studied with wheat near isogenic lines (NIL) carrying FHB-resistant and -susceptible alleles and with different parameters such as different tissues or time points. In the present study, we infected susceptible and MS wheat cultivars with Fg to analyze the gene expression alterations at 12 hai. As presented in our results, the number of differentially expressed transcripts was almost the same between susceptible and MS cultivars, but considering their functions, the gene profiles varied among each other. Therefore, defense response has already been started at 12 hai in both cultivars. Pritsch et al. (2000) previously reported that the macroconidia of Fg germinated within 6–12 hai on the tissues. It can be speculated that both of these two cultivars respond to pathogen but in different ways.

### PRs and detoxification genes play a role in early response to FHB

Upon pathogen infection, plant defense responses contain transcriptional regulation of a huge number of plant host genes. Global gene expression profiling suggests that induced disease resistance leads to the activation of partly overlapping sets of defense responses (Schenk et al., 2000; Conrath, 2011). Some of the pathogenesis-related (PR) proteins such as chitinases and glucanases which are capable of degrading the cell wall components of pathogens take place in plant response (Chen and Chen, 2002). It was previously reported that in Fg infected wheat the genes encoding *peroxidase, PR-1, PR-2, PR-3, PR-4*, and *PR-5* were induced after 6–12 h and reached the highest levels at 36–48 h (Pritsch et al., 2000, 2001). Desmond et al. (2008) reported in wheat infected by *F. pseudograminearum*, which is closely relative to Fg, that antimicrobial proteins like chitinase, β-1,3-glucanase, PR1, PR10, and thaumatin-like proteins were induced after 24 h. They also indicated that peroxidase involving reactive oxygen species (ROS) (Kawano, 2003) was induced (Desmond et al., 2008). In this study, it was observed that chitinase genes were expressed in both MS and susceptible cultivars, however the fold changes found in susceptible cv. “Gun-91” were approximately 2 times higher than those of MS cv. “Mizrak,” at 12 hai. Peroxidase types and their fold changes were also detected in varying levels in both cultivars. Inheritance of disease resistant genes should be differentiated in different cultivars of the same species, particularly those with defensive functions (Adhikari et al., 2007; Desmond et al., 2008). After inoculation of wheat with *M. graminicola*, Adhikari et al. (2007) reported that Chit, Pal, Per, and PR-1 were strongly induced as early as 3 h after inoculation of the two resistant cultivars, however, they indicated that susceptible cultivars showed slightly lower induction than resistant ones. As a result, some types of PR proteins had roles in defense response to pathogenic attack at 12 hai in wheat.

### Transcription factors and gene expression of early response to FHB

In response to external stimuli, complex changes occur in gene expression levels as up or down-regulation. The stimuli activate the primary response genes mediated by pre-existing signaling components such as TFs (Herschman, 1991). Therefore, transcriptional regulation of plant genes is a part of plant defense mechanism with an important role in induced plant disease resistance (Chen and Chen, 2002). We also compared the susceptible and MS cultivars at 12 hai with regards to the TFs. The most abundant ones (TFs) identified in this study were the members of *WRKY* and *bZIP*s families. It was well documented that *bZIP*s and *WRKY*s were two important plant transcription factor (TF) families regulating various developmental and stress-related processes (Llorca et al., 2014).

A total of 15 wheat *WRKY* cDNAs were obtained in wheat and the expression analysis of these genes showed that most of them were highly expressed in leaves (Wu et al., 2008). In our data, *WRKY*s were represented in a high number of transcripts. *WRKY TF 70* (Ta_S36162740) was found to be three times up-regulated in Mizrak at 12 hai compared to both its control and Gun-91 at 12 hai. It was previously reported that *WRKY*70 had a very important role in plant defense against pathogens by controlling the cross-talk of SA and JA signaling (Li et al., 2006; Besseau et al., 2012). Salicylic Acid (SA) pathway has a function in FHB resistance at early infection stage within about 12 hai (Ding et al., 2011). According to our data, JA and SA mediated signaling pathway responsive transcripts, *map kinase 5* (Ta_S12923226) and *mitogen-activated protein kinase kinase 6* (Ta_S12917674), were induced in Mizrak at 12 hai. It has been previously reported that mitogen-activated protein kinases, especially up-regulation of *MPK3* and *MPK6* which functions in signaling mechanisms, were found to increase immune response (Beckers and Conrath, 2007) (Beckers et al., 2009). These results were in agreement with previous reports (Taj et al., 2014). Considering these results, it was also evident that *WRKY*70 transcript was expressed by Mizrak at 12 hai in response to Fg infection. In Mizrak, *bZIP TF* (Ta_S17984954) was found to be repressed. It is likely that these two TFs are working antagonistically. The activity of the available bZIP monomers can be further regulated by phosphorylation. This kind of post translational modification can modify all the above-mentioned mechanisms controlling the TF function. As *bZIP*s, *WRKY*s activity can be modulated by phosphorylation through mitogen-activated protein kinase (MAPK) pathway (Lee et al., 2010; Llorca et al., 2014). In Mizrak cultivar at 12 hai, the transcripts related to protein kinase activity were mostly up-regulated (74%) and protein phosphorylation was as much as 75% compared to its control.

Analysis of the gene expression process clearly indicated that gene expressions were mostly up-regulated in Mizrak (MS), whereas they were down-regulated in Gun-91 (S). Previous studies have revealed that some environmental effects like pathogens can lead to alterations in translation (Bailey-Serres, 1999). The protein synthesis initiation in eukaryotes is facilitated by various translation initiation factors (eIFs). Translation initiation factor 3 (eIF3) complex stimulates binding of mRNA and methionyl-tRNA to the 40S ribosome with other initiation factors (Chaudhuri et al., 1999) and it was isolated from wheat germs (Checkley et al., 1981). One of the shared transcripts found in two cultivars was *eukaryotic translation initiation factor 3 subunit d* (Ta_S17890113), which was strongly expressed in Gun-91. Recent studies have displayed that eIF4E gene plays a role in resistance against viruses in barley (Robaglia and Caranta, 2006). Therefore, it can be suggested that this gene product may streamline the synthesis of some proteins, which have a role in pathogen defense mechanism.

### Transcript accumulation related carbohydrate metabolism upon Fg infection

It is necessary to find out how plants respond to stress, and which genes and pathways have functions upon stresses. We found 74 pathways in response to *Fusarium* and our analysis showed that starch and sucrose metabolism, purine metabolism and Glycolysis/Gluconeogenesis pathways were included as the most abundant transcripts. These annotations serve important clues for understanding early response of wheat against this pathogen. It was previously reported that alterations in carbohydrate metabolism could occur during the pathogen stress in plants (Gurkok et al., 2015) as well as in wheat (Wright et al., 1995; Kumar et al., 2014). On the other hand, it was mentioned earlier that there was a strong interaction between fungi and wheat considering both the antioxidant and glycolysis pathways (Zhou et al., 2006). Zhou et al. (2002) also reported enzymes of starch metabolism which affected *Fusarium* stress. In gene enrichment analysis, the number of transcripts taking part in carbohydrate metabolism was similar between MS and susceptible cultivars while the number of down-regulated transcripts was higher in Mizrak than in Gun-91. On the other hand, it is not clarified why carbohydrate metabolism or starch synthesis plays an important role on these cultivars.

## Conclusion

We performed transcriptome profiling of wheat leaves against the fungal pathogen Fg to explore the early response between susceptible and MS cultivars. It was observed that both MS and S cultivars were affected by Fg infection and activated their defense system, though in different ways, involving transcripts such as different PR proteins and TFs. The transcripts related to response to stimuli and metabolic process were the most affected processes by pathogenic stress. In addition, the number of binding activity and membrane-associated transcripts was higher in total. The KEGG pathway enrichment analyses showed that genes involved in carbohydrate and energy metabolisms were affected in early response. There were also highly expressed genes, which were not identified, hence new functional approaches should be adopted to clarify fungal pathogenity. Furthermore, considering the possibility of the genetic variation existed in wheat varieties with regards to resistance to Fg, our results may assist to find new markers for wheat breeders for improving FHB resistant varieties. Deep RNA sequencing will also be useful to generate new sequence variants related to resistance to FHB in wheat.

## Author contributions

TU and ME planned and organized the study. MT, TG, BI performed the microarray study, TG and GA performed MapMan and B2Go analyses. BI, TG performed qRT-PCR measurements. EIs and EIl inoculated wheat plants and isolated RNAs. ME, GA, MT, and TU drafted manuscript.

### Conflict of interest statement

The authors declare that the research was conducted in the absence of any commercial or financial relationships that could be construed as a potential conflict of interest.
